# 2,2′-[1,1′-(Ethyl­enedioxy­dinitrilo)diethyl­idyne]di-1-naphthol

**DOI:** 10.1107/S1600536809022508

**Published:** 2009-06-17

**Authors:** Wen-Kui Dong, Jian-chao Wu, Jian Yao, Li Li, Shang-sheng Gong

**Affiliations:** a School of Chemical and Biological Engineering, Lanzhou Jiaotong University, Lanzhou 730070, People’s Republic of China

## Abstract

The complete molecule of the title compound, C_26_H_24_N_2_O_4_, is generated by a crystallographic centre of inversion. There are two intra­molecular O—H⋯N hydrogen bonds. In the crystal structure, inter­molecular C—H⋯O hydrogen bonds result in zigzag chains.

## Related literature

For the applications of Shiff base ligands, see: Calligaris & Randaccio (1987 ▶) For the applications bis­oxime derivatives of salen-type compounds, see: Sun *et al.* (2004 ▶); Wang *et al.* (2007 ▶). For related structures, see: Dong *et al.* (2008*a* ▶,*b* ▶,*c* ▶);
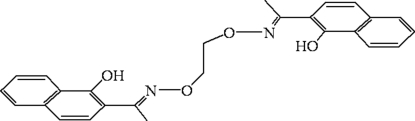


## Experimental

### 

#### Crystal data

C_26_H_24_N_2_O_4_
*M* *_r_* = 428.47
Monoclinic, 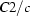

*a* = 12.6682 (18) Å
*b* = 9.3728 (15) Å
*c* = 18.335 (2) Å
β = 97.478 (2)°
*V* = 2158.6 (5) Å^3^
*Z* = 4
Mo *K*α radiation
μ = 0.09 mm^−1^
*T* = 298 K
0.39 × 0.37 × 0.13 mm

#### Data collection

Bruker SMART 1000 CCD area-detector diffractometer
Absorption correction: multi-scan (*SADABS*; Sheldrick, 1996 ▶) *T*_min_ = 0.966, *T*_max_ = 0.989
5220 measured reflections
1894 independent reflections
1027 reflections with *I* > 2σ(*I*)
*R*_int_ = 0.035

#### Refinement

*R*[*F* ^2^ > 2σ(*F* ^2^)] = 0.045
*wR*(*F* ^2^) = 0.122
*S* = 1.06
1894 reflections
145 parameters
H-atom parameters constrained
Δρ_max_ = 0.14 e Å^−3^
Δρ_min_ = −0.15 e Å^−3^

### 

Data collection: *SMART* (Siemens, 1996 ▶); cell refinement: *SAINT* (Siemens, 1996 ▶); data reduction: *SAINT*; program(s) used to solve structure: *SHELXS97* (Sheldrick, 2008 ▶); program(s) used to refine structure: *SHELXL97* (Sheldrick, 2008 ▶); molecular graphics: *SHELXTL* (Sheldrick, 2008 ▶); software used to prepare material for publication: *SHELXTL*.

## Supplementary Material

Crystal structure: contains datablocks global, I. DOI: 10.1107/S1600536809022508/hg2524sup1.cif

Structure factors: contains datablocks I. DOI: 10.1107/S1600536809022508/hg2524Isup2.hkl

Additional supplementary materials:  crystallographic information; 3D view; checkCIF report

## Figures and Tables

**Table 1:** Hydrogen-bond geometry (Å, °)

*D*—H⋯*A*	*D*—H	H⋯*A*	*D*⋯*A*	*D*—H⋯*A*
O2—H2⋯N1	0.82	1.84	2.562 (3)	146
C10—H10⋯O2^i^	0.93	2.63	3.446 (3)	146

Symmetry code: (i) 
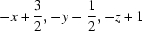
.

**Fig. 1.**
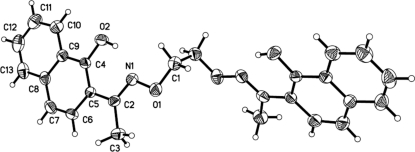
The molecular structure of the title compound with atom numbering scheme [Symmetry codes: -x+2, y, -z + 3/2]. Displacement ellipsoids for non-hydrogen atoms are drawn at the 30% probability level.

**Fig. 2.**
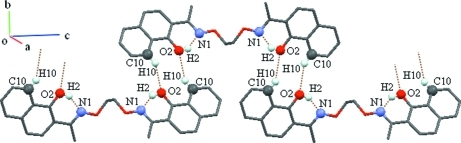
Part of the supramolecular structure of the title compound. Intra- and intermolecular hydrogen bonds are shown as dashed lines.

## supplementary crystallographic information

### Comment

There has been considerable interest in Schiff base ligand containing oxygen and imine nitrogen atoms due to their variety of applications, especially for catalysis and enzymatic reactions, magnetism, and supramolecular architectures (Calligaris & Randaccio, 1987). salen-type compounds and its bisoxime derivatives are a new class of multidentate ligand, which can be used as elemental building blocks for construction of supramolecular structures *via* intermolecular hydrogen bonding or short contact interaction (Sun *et al.*, 2004; Wang *et al.*, 2007). As an extension of our work (Dong *et al.*, 2008*a*; Dong *et al.*, 2008*b*) on the structural characterization of salen-type bisoxime compounds, here report the synthesis and structure of the title compound (Fig. 1).

The single-crystal structure of the title compound is built up by discrete C_26_H_24_N_2_O_4_ molecules, in which all bond lengths are in normal ranges. The molecule has a crystallographic twofold rotation axis (symmetry code: -*x*, *y*, 1/2 - *z*) and screw axis (symmetry code: 1/2 - *x*, 1/2 + *y*, 1/2 - *z*), and adopts a distorted E-configuration. This structure is similar to what was observed in our previously reported E-configuration compounds of 2,2'-[1,1'-Ethylenedioxybis(nitriloethylidyne)]diphenol (Wang *et al.*, 2007). The dihedral angle formed by the two naphthalene rings in each molecule of the title compound is about 43.20 °. There are two intramolecular hydrogen bonds, O2—H2···N1 (*d*(O2—H2) = 0.82 Å, d(H2···N1) = 1.84 Å, d(O2···N1) = 2.562 (3) Å, <O2—H2···N1 = 146°). Besides in the crystal structure, four intermolecular hydrogen bonds, C10—H10···O2 (*d*(C10—H10) = 0.93 Å, d(H10···O2) = 2.63 Å, d(C10···O2) = 3.446 (3) Å, <C10—H10···O2 = 146°), link two other molecules into infinite zigzag supramolecular structure (Fig. 2).

### Experimental

2,2'-[1,1'-Ethylenedioxybis(nitriloethylidyne)]dinaphthol was synthesized according to an analogous method reported earlier (Dong *et al.*, 2008*c*). To an ethanol solution (5 ml) of 2-acetyl-1-naphthol (392.1 mg, 2.10 mmol) was added dropwise an ethanol solution (3 ml) of 1,2-bis(aminooxy)ethane (96.2 mg, 1.04 mmol). The mixture solution was stirred at 328–433 K for 72 h. After cooling to room temperature, the precipitate was filtered off, and washed successively three times with ethanol. The product was dried *in vacuo* and purified by recrystallization from ethanol to yield 313.7 mg (Yield, 70.1%) of powder; m.p. 471–472 K. Colorless block-like single crystals suitable for X-ray diffraction studies were obtained by slow evaporation from a solution of dichloromethane of 2,2'-[1,1'-ethylenedioxybis(nitriloethylidyne)]dinaphthol at room temperature for about three weeks. Anal. Calc. for C_26_H_24_N_2_O_4_: C, 73.28; H, 5.92; N, 6.33; Found: C, 73.25; H, 5.97; N, 6.29.

### Refinement

Non-H atoms were refined anisotropically. H atoms were treated as riding atoms with distances C—H = 0.97 (CH_2_), C—H = 0.96 (CH_3_), 0.93 Å (CH), 0.82 Å (OH), and *U*_iso_(H) = 1.2 *U*_eq_(C) and 1.5 *U*_eq_(O).

### Crystal data

**Table:**

C_26_H_24_N_2_O_4_	*F*(000) = 904
*M**_r_* = 428.47	*D*_x_ = 1.318 Mg m^−^^3^
Monoclinic, *C*2/*c*	Mo *K*α radiation, λ = 0.71073 Å
Hall symbol: -C 2yc	Cell parameters from 1610 reflections
*a* = 12.6682 (18) Å	θ = 2.2–26.7°
*b* = 9.3728 (15) Å	µ = 0.09 mm^−^^1^
*c* = 18.335 (2) Å	*T* = 298 K
β = 97.478 (2)°	Block-shaped, colorless
*V* = 2158.6 (5) Å^3^	0.39 × 0.37 × 0.13 mm
*Z* = 4	

### Data collection

**Table:**

Bruker SMART 1000 CCD area-detector diffractometer	1894 independent reflections
Radiation source: fine-focus sealed tube	1027 reflections with *I* > 2σ(*I*)
graphite	*R*_int_ = 0.035
φ and ω scans	θ_max_ = 25.0°, θ_min_ = 2.2°
Absorption correction: multi-scan (*SADABS*; Sheldrick, 1996)	*h* = −15→14
*T*_min_ = 0.966, *T*_max_ = 0.989	*k* = −11→9
5220 measured reflections	*l* = −18→21

### Refinement

**Table:**

Refinement on *F*^2^	Primary atom site location: structure-invariant direct methods
Least-squares matrix: full	Secondary atom site location: difference Fourier map
*R*[*F*^2^ > 2σ(*F*^2^)] = 0.045	Hydrogen site location: inferred from neighbouring sites
*wR*(*F*^2^) = 0.122	H-atom parameters constrained
*S* = 1.06	*w* = 1/[σ^2^(*F*_o_^2^) + (0.037*P*)^2^ + 1.5505*P*] where *P* = (*F*_o_^2^ + 2*F*_c_^2^)/3
1894 reflections	(Δ/σ)_max_ < 0.001
145 parameters	Δρ_max_ = 0.14 e Å^−^^3^
0 restraints	Δρ_min_ = −0.15 e Å^−^^3^

### Special details

**Table:**

Geometry. All e.s.d.'s (except the e.s.d. in the dihedral angle between two l.s. planes) are estimated using the full covariance matrix. The cell e.s.d.'s are taken into account individually in the estimation of e.s.d.'s in distances, angles and torsion angles; correlations between e.s.d.'s in cell parameters are only used when they are defined by crystal symmetry. An approximate (isotropic) treatment of cell e.s.d.'s is used for estimating e.s.d.'s involving l.s. planes.
Refinement. Refinement of *F*^2^ against ALL reflections. The weighted *R*-factor *wR* and goodness of fit *S* are based on *F*^2^, conventional *R*-factors *R* are based on *F*, with *F* set to zero for negative *F*^2^. The threshold expression of *F*^2^ > σ(*F*^2^) is used only for calculating *R*-factors(gt) *etc*. and is not relevant to the choice of reflections for refinement. *R*-factors based on *F*^2^ are statistically about twice as large as those based on *F*, and *R*- factors based on ALL data will be even larger.

### Fractional atomic coordinates and isotropic or equivalent isotropic displacement parameters (Å^2^)

**Table:**

	*x*	*y*	*z*	*U*_iso_*/*U*_eq_	
N1	0.97510 (17)	0.0879 (2)	0.61829 (11)	0.0528 (6)	
O1	1.04967 (14)	0.11327 (19)	0.68140 (9)	0.0610 (5)	
O2	0.84279 (14)	−0.06506 (17)	0.53429 (9)	0.0620 (6)	
H2	0.8831	−0.0456	0.5717	0.093*	
C1	1.0479 (2)	−0.0049 (3)	0.73030 (14)	0.0567 (7)	
H1A	1.1113	−0.0024	0.7661	0.068*	
H1B	1.0493	−0.0929	0.7025	0.068*	
C2	0.9707 (2)	0.1902 (3)	0.57048 (14)	0.0500 (7)	
C3	1.0376 (2)	0.3226 (3)	0.58230 (16)	0.0714 (9)	
H3A	1.0938	0.3072	0.6220	0.107*	
H3B	0.9941	0.4009	0.5942	0.107*	
H3C	1.0678	0.3444	0.5382	0.107*	
C4	0.83740 (19)	0.0469 (3)	0.48738 (13)	0.0464 (6)	
C5	0.89572 (19)	0.1710 (2)	0.50274 (12)	0.0469 (6)	
C6	0.8818 (2)	0.2819 (3)	0.44918 (15)	0.0610 (8)	
H6	0.9202	0.3660	0.4582	0.073*	
C7	0.8146 (2)	0.2692 (3)	0.38547 (16)	0.0676 (8)	
H7	0.8065	0.3454	0.3527	0.081*	
C8	0.7568 (2)	0.1424 (3)	0.36816 (14)	0.0552 (7)	
C9	0.7676 (2)	0.0289 (3)	0.42035 (13)	0.0490 (7)	
C10	0.7106 (2)	−0.0991 (3)	0.40331 (15)	0.0632 (8)	
H10	0.7163	−0.1737	0.4370	0.076*	
C11	0.6470 (2)	−0.1137 (4)	0.33734 (18)	0.0774 (9)	
H11	0.6097	−0.1982	0.3266	0.093*	
C12	0.6378 (2)	−0.0025 (4)	0.28598 (17)	0.0794 (10)	
H12	0.5957	−0.0146	0.2409	0.095*	
C13	0.6896 (2)	0.1227 (4)	0.30122 (15)	0.0707 (9)	
H13	0.6807	0.1966	0.2671	0.085*	

### Atomic displacement parameters (Å^2^)

**Table:**

	*U*^11^	*U*^22^	*U*^33^	*U*^12^	*U*^13^	*U*^23^
N1	0.0581 (14)	0.0524 (13)	0.0491 (13)	−0.0069 (11)	0.0112 (11)	−0.0060 (11)
O1	0.0618 (12)	0.0706 (13)	0.0511 (11)	−0.0170 (10)	0.0085 (9)	−0.0068 (9)
O2	0.0768 (13)	0.0463 (10)	0.0591 (12)	−0.0120 (9)	−0.0055 (10)	0.0078 (9)
C1	0.0569 (18)	0.0582 (17)	0.0548 (17)	0.0022 (13)	0.0063 (13)	−0.0011 (14)
C2	0.0553 (16)	0.0410 (14)	0.0572 (17)	−0.0059 (13)	0.0205 (13)	−0.0099 (13)
C3	0.085 (2)	0.0560 (18)	0.076 (2)	−0.0235 (16)	0.0195 (17)	−0.0080 (15)
C4	0.0559 (17)	0.0381 (14)	0.0474 (15)	0.0028 (12)	0.0152 (13)	0.0019 (12)
C5	0.0582 (16)	0.0398 (14)	0.0458 (15)	−0.0022 (13)	0.0176 (13)	−0.0033 (12)
C6	0.080 (2)	0.0431 (15)	0.0637 (19)	−0.0059 (15)	0.0249 (16)	0.0047 (14)
C7	0.086 (2)	0.0622 (19)	0.0587 (19)	0.0079 (17)	0.0262 (17)	0.0161 (15)
C8	0.0593 (18)	0.0602 (18)	0.0491 (16)	0.0143 (15)	0.0180 (14)	0.0030 (14)
C9	0.0485 (16)	0.0489 (16)	0.0509 (16)	0.0069 (13)	0.0117 (13)	−0.0021 (13)
C10	0.0617 (18)	0.0606 (18)	0.0659 (19)	−0.0008 (15)	0.0034 (16)	−0.0052 (15)
C11	0.064 (2)	0.087 (2)	0.079 (2)	−0.0027 (18)	−0.0008 (18)	−0.0190 (19)
C12	0.061 (2)	0.116 (3)	0.059 (2)	0.016 (2)	0.0015 (16)	−0.012 (2)
C13	0.065 (2)	0.094 (2)	0.0537 (19)	0.0235 (19)	0.0121 (16)	0.0088 (17)

### Geometric parameters (Å, °)

**Table:**

N1—C2	1.295 (3)	C5—C6	1.425 (3)
N1—O1	1.415 (2)	C6—C7	1.358 (3)
O1—C1	1.427 (3)	C6—H6	0.9300
O2—C4	1.353 (3)	C7—C8	1.410 (4)
O2—H2	0.8200	C7—H7	0.9300
C1—C1^i^	1.490 (5)	C8—C13	1.412 (4)
C1—H1A	0.9700	C8—C9	1.426 (3)
C1—H1B	0.9700	C9—C10	1.413 (3)
C2—C5	1.473 (3)	C10—C11	1.370 (3)
C2—C3	1.504 (3)	C10—H10	0.9300
C3—H3A	0.9600	C11—C12	1.400 (4)
C3—H3B	0.9600	C11—H11	0.9300
C3—H3C	0.9600	C12—C13	1.355 (4)
C4—C5	1.387 (3)	C12—H12	0.9300
C4—C9	1.428 (3)	C13—H13	0.9300
			
C2—N1—O1	113.2 (2)	C7—C6—C5	122.5 (3)
N1—O1—C1	108.64 (18)	C7—C6—H6	118.8
C4—O2—H2	109.5	C5—C6—H6	118.8
O1—C1—C1^i^	112.64 (18)	C6—C7—C8	121.1 (3)
O1—C1—H1A	109.1	C6—C7—H7	119.5
C1^i^—C1—H1A	109.1	C8—C7—H7	119.5
O1—C1—H1B	109.1	C7—C8—C13	122.9 (3)
C1^i^—C1—H1B	109.1	C7—C8—C9	118.4 (2)
H1A—C1—H1B	107.8	C13—C8—C9	118.7 (3)
N1—C2—C5	116.6 (2)	C10—C9—C8	118.9 (2)
N1—C2—C3	122.7 (2)	C10—C9—C4	122.1 (2)
C5—C2—C3	120.8 (2)	C8—C9—C4	118.9 (2)
C2—C3—H3A	109.5	C11—C10—C9	120.3 (3)
C2—C3—H3B	109.5	C11—C10—H10	119.8
H3A—C3—H3B	109.5	C9—C10—H10	119.8
C2—C3—H3C	109.5	C10—C11—C12	120.5 (3)
H3A—C3—H3C	109.5	C10—C11—H11	119.8
H3B—C3—H3C	109.5	C12—C11—H11	119.8
O2—C4—C5	122.7 (2)	C13—C12—C11	120.7 (3)
O2—C4—C9	115.4 (2)	C13—C12—H12	119.7
C5—C4—C9	121.9 (2)	C11—C12—H12	119.7
C4—C5—C6	117.2 (2)	C12—C13—C8	120.9 (3)
C4—C5—C2	122.8 (2)	C12—C13—H13	119.6
C6—C5—C2	120.1 (2)	C8—C13—H13	119.6
			
C2—N1—O1—C1	179.8 (2)	C6—C7—C8—C9	2.2 (4)
N1—O1—C1—C1^i^	−75.0 (3)	C7—C8—C9—C10	−179.5 (2)
O1—N1—C2—C5	179.31 (19)	C13—C8—C9—C10	−0.1 (4)
O1—N1—C2—C3	−0.8 (3)	C7—C8—C9—C4	−0.9 (3)
O2—C4—C5—C6	−179.2 (2)	C13—C8—C9—C4	178.5 (2)
C9—C4—C5—C6	1.1 (3)	O2—C4—C9—C10	−1.9 (3)
O2—C4—C5—C2	1.7 (4)	C5—C4—C9—C10	177.9 (2)
C9—C4—C5—C2	−178.1 (2)	O2—C4—C9—C8	179.6 (2)
N1—C2—C5—C4	−4.3 (3)	C5—C4—C9—C8	−0.7 (3)
C3—C2—C5—C4	175.8 (2)	C8—C9—C10—C11	0.6 (4)
N1—C2—C5—C6	176.6 (2)	C4—C9—C10—C11	−177.9 (2)
C3—C2—C5—C6	−3.3 (4)	C9—C10—C11—C12	0.1 (4)
C4—C5—C6—C7	0.2 (4)	C10—C11—C12—C13	−1.5 (5)
C2—C5—C6—C7	179.3 (2)	C11—C12—C13—C8	2.1 (5)
C5—C6—C7—C8	−1.8 (4)	C7—C8—C13—C12	178.1 (3)
C6—C7—C8—C13	−177.2 (3)	C9—C8—C13—C12	−1.3 (4)

Symmetry codes: (i) −*x*+2, *y*, −*z*+3/2.

### Hydrogen-bond geometry (Å, °)

**Table:**

*D*—H···*A*	*D*—H	H···*A*	*D*···*A*	*D*—H···*A*
O2—H2···N1	0.82	1.84	2.562 (3)	146
C10—H10···O2^ii^	0.93	2.63	3.446 (3)	146

Symmetry codes: (ii) −*x*+3/2, −*y*−1/2, −*z*+1.
